# Interventions to Slow Aging in Humans: Are We Ready?

**DOI:** 10.1111/acel.12338

**Published:** 2015-04-22

**Authors:** Valter D Longo, Adam Antebi, Andrzej Bartke, Nir Barzilai, Holly M Brown-Borg, Calogero Caruso, Tyler J Curiel, Rafael de Cabo, Claudio Franceschi, David Gems, Donald K Ingram, Thomas E Johnson, Brian K Kennedy, Cynthia Kenyon, Samuel Klein, John J Kopchick, Guenter Lepperdinger, Frank Madeo, Mario G Mirisola, James R Mitchell, Giuseppe Passarino, Karl L Rudolph, John M Sedivy, Gerald S Shadel, David A Sinclair, Stephen R Spindler, Yousin Suh, Jan Vijg, Manlio Vinciguerra, Luigi Fontana

**Affiliations:** 1 Davis School of Gerontology and Department of Biological Sciences, Longevity Institute, University of Southern CaliforniaLos Angeles, CA, 90089, USA; 2 IFOM, FIRC Institute of Molecular OncologyVia Adamello 16, 20139, Milano, Italy; 3 Max Planck Institute for Biology of AgeingJoseph Stelzmann Strasse 9b, 50931, Koeln, Germany; 4 Department of Internal Medicine, Southern Illinois University-School of MedicineSpringfield, IL, 62794, USA; 5 Institute for Aging Research, Albert Einstein College of Medicine1300 Morris Park Ave, Bronx, NY, 10461, USA; 6 Department of Basic Sciences, University of North Dakota School of Medicine and Health SciencesGrand Forks, ND, 58203, USA; 7 Immunosenescence Unit, Department of Pathobiology and Medical and Forensic Biotechnologies, University of PalermoPalermo, Italy; 8 Department of Medicine, University of Texas Health Science CenterSan Antonio, TX, USA; 9 Experimental Gerontology Section, TGB, NIA, NIH251 Bayview Blvd. Suite 100/Room 9C218, Baltimore, MD, 21224, USA; 10 DIMES-Department of Specialty, Diagnostic and Experimental MedicineVia S. Giacomo, 12, I-40126, Bologna, Italy; 11 Department of Genetics, Evolution and Environment, Institute of Healthy Ageing, University College LondonThe Darwin Building, Gower Street, London, WC1E 6BT, UK; 12 Nutritional Neuroscience and Aging Laboratory, Pennington Biomedical Research Center, Louisiana State University System6400 Perkins Road, Baton Rouge, LA, 70809, USA; 13 Institute for Behavioral Genetics, University of Colorado at BoulderBox 447, Boulder, CO, 80309, USA; 14 Buck Institute for Research on AgingNovato, CA, USA; 15 Department of Biochemistry and Biophysics, Mission Bay Genentech Hall, University of California600 16th Street, Room S312D, San Francisco, CA, 94158-2517, USA; 16 Center for Human Nutrition, Washington University School of MedicineCampus Box 8031, 660 South Euclid Avenue, St. Louis, MO, 63110, USA; 17 Department of Biomedical Sciences, Edison Biotechnology Institute, Heritage College of Osteopathic Medicine, Ohio UniversityAthens, OH, 45701, USA; 18 Institut für Alternsforschung, Universität InnsbruckRennweg 10, A-6020, Innsbruck, Austria; 19 Institute of Molecular Biosciences, NAWI Graz, University of GrazHumboldtstr. 50, Graz, 8010, Austria; 20 BioTechMed GrazHumboldtstr. 50, Graz, 8010, Austria; 21 Dipartimento di Biopatologia e Biotecnologie mediche, Universita’ di PalermoVia Divisi 83, 90133, Palermo, Italy; 22 Department of Genetics and Complex Diseases, Harvard School of Public HealthBoston, MA, 02115, USA; 23 Department of Biology, Ecology and Earth Science, University of Calabria87036, Rende, Italy; 24 Leibniz Institute for Age Research, Fritz Lipmann InstituteD-07745, Jena, Germany; 25 Laboratories for Molecular Medicine, Department of Molecular Biology, Cell Biology and Biochemistry, Brown University70 Ship Street, Providence, RI, 02903, USA; 26 Department of Pathology, Yale University School of MedicineNew Haven, CT, 06520, USA; 27 Department of Genetics, Yale University School of MedicineNew Haven, CT, 06520, USA; 28 Laboratory for Ageing Research, Department of Pharmacology, School of Medical Sciences, UNSW AustraliaSydney, NSW, Australia; 29 Australia Glenn Labs for the Biological Mechanisms of Aging, Department of Genetics, Harvard Medical SchoolBoston, MA, Australia; 30 Department of Biochemistry, University of California at RiversideRiverside, CA, USA; 31 Department of Genetics, Albert Einstein College of MedicineBronx, NY, USA; 32 Department of Medicine, Diabetes Research and Training Center, Albert Einstein College of MedicineBronx, NY, USA; 33 Institute for Aging Research, Diabetes Research and Training Center, Albert Einstein College of MedicineBronx, NY, USA; 34 Department of Genetics, Albert Einstein Medical Center1301 Morris Park Avenue, Bronx, NY, USA; 35 Division of Medicine, University College London (UCL) – Institute for Liver and Digestive Health, Royal Free HospitalLondon, UK; 36 Department of Medicine, Washington University School of MedicineSt. Louis, MO, USA; 37 Department of Clinical and Experimental Sciences, Brescia UniversityBrescia, Italy; 38 CEINGE Biotecnologie AvanzateNapoli, Italy

**Keywords:** aging, anti-aging, centenarians, longevity regulation, dietary restriction, lifespan studies, longevity gene

## Abstract

The workshop entitled ‘Interventions to Slow Aging in Humans: Are We Ready?’ was held in Erice, Italy, on October 8–13, 2013, to bring together leading experts in the biology and genetics of aging and obtain a consensus related to the discovery and development of safe interventions to slow aging and increase healthy lifespan in humans. There was consensus that there is sufficient evidence that aging interventions will delay and prevent disease onset for many chronic conditions of adult and old age. Essential pathways have been identified, and behavioral, dietary, and pharmacologic approaches have emerged. Although many gene targets and drugs were discussed and there was not complete consensus about all interventions, the participants selected a subset of the most promising strategies that could be tested in humans for their effects on healthspan. These were: (i) dietary interventions mimicking chronic dietary restriction (periodic fasting mimicking diets, protein restriction, etc.); (ii) drugs that inhibit the growth hormone/IGF-I axis; (iii) drugs that inhibit the mTOR–S6K pathway; or (iv) drugs that activate AMPK or specific sirtuins. These choices were based in part on consistent evidence for the pro-longevity effects and ability of these interventions to prevent or delay multiple age-related diseases and improve healthspan in simple model organisms and rodents and their potential to be safe and effective in extending human healthspan. The authors of this manuscript were speakers and discussants invited to the workshop. The following summary highlights the major points addressed and the conclusions of the meeting.

## Introduction

Human aging and age-associated diseases are emerging as among the greatest challenges and financial burdens faced by developed and developing countries (Christensen *et al*., 2009) (http://esa.un.org/wpp/documentation/pdf/WPP2010_Volume-I_Comprehensive-Tables.pdf). Although average life expectancy has increased dramatically in the last 100 years, this has not been accompanied by an equivalent increase in healthy life expectancy, which has been termed healthspan (Hung *et al*., 2011). Research related to longevity extension has traditionally been viewed with skepticism and with concerns that it could lead to an increase in the size of the elderly population and the prevalence of diseases associated with aging. However, studies in a wide range of organisms have demonstrated that major lifespan extension is accompanied by reduced or delayed morbidity in most cases (Fontana *et al*., 2010). Data from experimental studies in invertebrates and rodents have consistently shown that both chronic dietary restriction (DR) and mutations in nutrient and growth signaling pathways can extend longevity by 30–50%. These interventions can also lower the prevalence of age-related loss of function and multiple diseases, including tumors, cardiovascular disease, and neurodegeneration (Fontana *et al*., 2010). DR protects against diabetes, cancer, cardiovascular disease, sarcopenia, and neurodegeneration of certain brain regions in rhesus monkeys and also extends lifespan (Mattison *et al*., 2012; Colman *et al*., 2014). In humans, long-term DR causes several metabolic and molecular changes that protect against age-related pathologies, including changes in markers for type 2 diabetes, hypertension, cardiovascular disease, cancer, and dementia (Cava & Fontana, 2013). Moreover, chronic DR ameliorates the expected age-associated alterations in myocardial stiffness, autonomic function, and skeletal muscle gene expression changes (Mattison *et al*., 2012; Cava & Fontana, 2013; Colman *et al*., 2014). However, the duration and severity of the DR regimen that is required for optimal benefits is not feasible for most people and is likely to be associated with undesirable side effects. Therefore, the focus of this consensus meeting in Erice was on less drastic dietary interventions and drugs that target nutrient-response pathways and mimic the effects of DR, but that are practical, realistic, and safe.

There was a general consensus among this panel of experts on the following points: (i) aging can be slowed by many interventions; (ii) slowing aging typically delays or prevents a range of chronic diseases of old age; (iii) dietary, nutraceutical, and pharmacologic interventions that modulate relevant intracellular signaling pathways and can be considered for human intervention have been identified. Additional potential targets will continue to emerge as research progresses; and (iv) it is now necessary to cautiously proceed to test these interventions in humans. Based on a vote taken on the last day of the workshop, the strategies believed to be most promising by the panel of invited experts and authors of this manuscript are as follows:

1. Pharmacological inhibition of the GH/IGF-1 axis
2. Protein restriction and Fasting Mimicking Diets
3. Pharmacological inhibition of the TOR -S6K pathway
4. Pharmacological regulation of certain sirtuin proteins and the use of spermidine and other epigenetic modulators
5. Pharmacological inhibition of inflammation
6. Chronic metformin use

These choices were based in part on: (i) consistent evidence for their pro-longevity effects in simple model organisms and rodents; (ii) evidence for their ability to prevent or delay multiple age-related diseases and conditions; and/or (iii) clinical evidence for their safety in small mammals and/or nonhuman primates. Below, we present the salient points related to each of the promising strategies. Other ideas discussed at the meeting are described elsewhere (Gems, 2014).

## Biomarkers to assess the efficacy of interventions

The successful development and implementation of interventions to promote healthspan will require the use of aging biomarkers. Biomarkers are defined here as biological characteristics that can be objectively measured and evaluated as indicators of age-related normal and pathogenic processes. In practice, such biomarkers should aid in diagnosing individual aging phenotypes, predicting the progression of these phenotypes, selecting possible interventions, and evaluating the effects and outcomes of such interventions. Biomarkers are key for the development, testing, and implementation of interventions in aging because of the great difficulty in performing clinical trials. Indeed, biomarkers of disease are often an aid in clinical trials, For example, to stratify patients so that only those likely to respond to a particular therapy receive it. Interventions in aging require biomarkers for all the same reasons, and also as surrogates for determining the efficacy and safety of interventions that often require very long time scales. There are three categories of such biomarkers that should be distinguished:

### Biomarkers of critical pro-aging mechanisms

These biomarkers are needed to define the parameters that should be targeted in interventions. A disease can be defined as a disorder or abnormality of structure or function. Some have argued that aging cannot be abnormal because everyone suffers from it, which has led to the reluctance to accept aging as the equivalent of a disease. This and the extended period of time that is required to evaluate the effect of interventions on aging essentially constrain clinical trials to testing the efficacy of experimental interventions. Hence, biomarkers are needed to predict the effect of interventions over the long term.

Despite the great progress in our understanding of how lifespan can be modulated in experimental animals, human aging itself remains less well understood. Biomarkers need to be defined for those processes deemed critical for healthy human aging. To develop such biomarkers, integrated animal–human studies are needed with an important role for systems biology to understand better the molecular basis of the critical pro-aging processes targeted for intervention and the mechanistic rationale of potential interventions. Data on epigenetic changes and other ‘omics’ level alterations with potential significance for critical pro-aging molecular pathways are important to collect.

### Companion biomarkers

For each of the experimental interventions deemed promising enough for limited human testing, biomarkers need to be developed that are able to: (i) identify individuals who would benefit from the treatment; and (ii) help understand and assess the molecular target(s) of the agent being studied. The high failure rate of new therapies tested in clinical development accentuates the critical importance of discovering biomarker readouts that can predict efficacy.

### Safety biomarkers

The development of 30% of new drug candidates is halted due to unforeseen toxicity and other adverse side effects in clinical studies. This is especially relevant for interventions in aging, which cannot be tested easily in clinical trials and need to be applied over extended periods of time. Accordingly, greater emphasis must be placed on the development and use of safety biomarkers in studies of interventions in aging. In this respect, it is possible to profit from safety biomarkers that have been developed and accepted over the years, for example, to detect toxicity in the kidney and liver.

Companion biomarkers and safety biomarkers are not specific to aging or age-related disease. The challenge to the field is to develop a consensus panel of biomarkers of aging that can be used in clinical trials, that is, biomarkers in category 1 above. The following is a list of potential biomarkers or methods to assess biological age discussed at the workshop. It does not represent a comprehensive listing of all possible biomarkers, but a list of potential markers and methods to assess biological age and the risk of developing age-related diseases suggested by the participants and discussed at the meeting.

1. Standard frailty measurements (e.g., walking speed at older ages, hand grip, VO_2max_)
2. Insulin, insulin resistance, fasting blood glucose + GTT, HbA1c, adiponectin, DEXA (abdominal adiposity)
3. LDL, HDL, blood pressure, pulse wave velocity, intima media thickness, left ventricular diastolic function/pressure
4. Inflammatory markers (e.g., CRP, IL-6, TNF-α)
5. Cognitive performance (cognitive tests, fMRIs)
6. Lymphocyte number, lymphoid/myeloid ratio
7. IGF-I, T3
8. Epigenetic profile (e.g., DNA methylation)
9. Renal clearance (age 60–90)

## Dietary interventions mimicking calorie restriction

### Intermittent and prolonged fasting

Fasting is the most extreme of the DR interventions because it requires the complete elimination of nutrients. The best-characterized form of fasting evaluated in both rodent and human studies is intermittent or alternate-day fasting (IF or ADF), which involves the feeding on every other day for long periods of time (Trepanowski *et al*., 2011). Prolonged fasting (PF), in which water but not food is consumed for two or more consecutive days, is also becoming a well-investigated intervention, particularly in rodent models and lower eukaryotes (Longo & Mattson, 2014). The mechanisms of action of fasting are best understood in the yeast *Saccharomyces cerevisiae,* in which a switch from glucose-containing medium to water causes the downregulation of the Tor–S6K and Ras–adenylate cyclase–PKA pathways, and the consequent activation of the stress resistance transcription factors Msn2/4 and Gis1, which regulate many protective and metabolic genes (Wei *et al*., 2008). These changes, similar to those observed in starved worms and mice, promote resistance to both multiple toxins and longevity extension. In mice, fasting reduces circulating IGF-I and causes downregulation of PI3K–AKT, Tor–S6K, and adenylate cyclase–PKA signaling, which, analogously to yeast, results in the activation of multiple transcription factors including members of the FOXO family of forkhead transcription factors (Cheng *et al*., 2014).

Different types of PF or IF have been shown to extend the lifespan of bacteria, worms, and rodents (Longo & Mattson, 2014). In mouse models, IF helps to prevent or delay the progression of myocardial infarction, diabetes, stroke, Alzheimer’s disease, and Parkinson’s disease (Longo & Mattson, 2014; Mattson, 2014). Similarly, PF protects mice against the adverse effects of chemotherapy and ischemia/reperfusion-mediated toxicity as well as cancer progression and promotes stem cell-dependent regeneration and immune system rejuvenation in old animals (Mauro *et al*., 2014, Safdie *et al*., 2009, Longo & Mattson 2014 Cheng *et al*., 2014; Levine *et al*., 2014). Although the effect of chronic cycles of PF on healthspan is not known, these studies point to PF, which could be carried out in humans as infrequently as once a month or less, as a potent inducer of protective systems and a potential alternative to chronic CR and IF.

A number of PF and IF clinical studies in humans are yielding very promising results in support of the possibility that they are sufficiently safe and effective to be considered for long-term clinical trials focused on healthspan (Longo & Mattson, 2014). Among the initial but solid evidence for efficacy is that PF followed by a vegetarian diet reduces both inflammation and pain in patients with rheumatoid arthritis (Michalsen *et al*., 2005). Preliminary studies suggest that PF may also decrease adverse effects of chemotherapy in humans (Safdie *et al*., 2009), a finding now being tested in multiple larger randomized clinical trials. Part of the protective effects of fasting against aging and disease may be mediated by the reduction in IGF-1, glucose, and insulin (see following sections).

The health effects of IF in humans have been investigated more extensively than those of PF. For example, 3 weeks of alternate-day fasting reduces body weight, body fat, and plasma insulin concentrations in both men and women (Heilbronn *et al*., 2005), while a diet of 500–600 calories on 2 of 7 days per week induces loss of abdominal fat, improves insulin sensitivity, and reduces blood pressure (Harvie *et al*., 2010).

PF and IF have few adverse effects, but could be dangerous for subjects of very low BMI, those who are frail and old, and patients with diabetes receiving insulin or insulin-like drugs. Thus, the lack of medical supervision in subjects undergoing IF or PF could result in severe adverse effects. For example, the insulin-sensitizing effects of PF can cause severe hypoglycemia and even death in patients with diabetes treated with insulin. Although major adverse effects caused by IF or PF are rare and usually reversible, these examples should underscore the potency of these interventions and their potential to cause remarkably global and beneficial effects, but also cause detrimental effects if the underlying mechanism of action are not well understood and they are not properly tested clinically and implemented. These issues point to the need for the identification and preclinical/clinical testing of fasting mimicking diets that match or even surpass the effects of fasting while minimizing the burden and adverse effects associated with water-only regimens.

### Protein restriction or selective amino acid restriction

The benefits attributed to reduced calorie intake could be due, in part, to the concomitant restriction of proteins or individual essential amino acids. Restriction of calories in the form of protein contributes to the benefits of DR on animal longevity (Gallinetti *et al*., 2013; Mirzaei *et al*., 2014). Restriction of individual essential amino acids, including methionine and tryptophan, can also extend longevity (Spindler, 2009). Notably it remains unclear whether the underlying mechanisms responsible for increased longevity overlap with those of other DR regimens. It is also not known whether there is some specificity to the effects of restriction of different essential amino acids.

Amino acid levels are sensed by at least two evolutionarily conserved mechanisms: one involving GCN2 (general control nonderepressible 2) and the other involving mTOR. In mammals, mTOR is activated by amino acids, particularly leucine, while GCN2 is activated by the absence of many individual amino acids. Mechanisms of protection downstream of GCN2 activation and mTOR repression subsequent to protein/amino acid starvation remain largely unknown. However, the transcription factor ATF4 is stabilized upon GCN2 activation and could be a key mediator in a variety of extended longevity models, including hypopituitary dwarf models and methionine restriction (Li *et al*., 2014).

The major factor controlling the longevity benefits of protein restriction in multiple species is the ratio of dietary protein to other macronutrient calorie sources (carbohydrates, fats), which is likely to affect aging in part through the regulation of mTORC1 signaling (Solon-Biet *et al*., 2014). Longevity extension by methionine restriction in yeast requires GCN2 (Wu *et al*., 2013), but other mechanisms including autophagy (Ruckenstuhl *et al*., 2014) and retrograde signaling from mitochondria (Johnson & Johnson, 2014) are implicated. In yeast, deficiencies in serine, threonine and valine, extend longevity by a mechanism involving the down-regulation of orthologs of mammalian PDK and Tor-S6K proteins (Mirisola, *et al*., 2014). In flies, reduced dietary methionine also extends longevity although the mechanism responsible for this effect is poorly understood (Lee *et al*., 2014). It has been shown that GH signaling is necessary to discriminate levels of dietary methionine in mice (Brown-Borg *et al*. 2014). In rodents, protein/amino acid restriction also offers health benefits in models of acute stress and chronic disease. Eliminating tryptophan from the diet for 1 week increases the resistance to the acute stress of ischemia/reperfusion injury in liver and kidney and this depends on GCN2 (Peng *et al*., 2012). However, GCN2 is no longer necessary for this beneficial effect when animals are also deprived of total protein. Instead, mTORC1 downregulation is key to stress resistance in this case. This effect could be mediated in part through the negative action of mTORC1 on insulin sensitivity, such that its downregulation allows for increased pro-survival signaling after reperfusion (Harputlugil *et al*., 2014).

Benefits of short-term protein deprivation also include protection from intimal hyperplasia (Mauro *et al*., 2014). Similarly, cycles of protein deprivation lasting 1 week, followed by a week of *ad libitum* access to a complete diet, protect against Tau phosphorylation in a mouse model of Alzheimer’s disease (Parrella *et al*., 2013). Finally, protein restriction has beneficial effects on metabolism, activating a fasting-like response that requires both GCN2 and PPAR-alpha (peroxisome proliferator-activated receptor-alpha), and that causes an increase in the fasting hormone FGF21 (Laeger *et al*., 2014).

To date, very few studies have been performed in humans on the potential beneficial effects of protein and/or amino acid restriction on aging processes or age-associated chronic diseases (Cavuoto & Fenech, 2012; Mirzaei *et al*., 2014). In agreement with mouse studies (Solon-Biet *et al*., 2014), the lowest protein intake was associated with reduced risk for cancer incidence and overall mortality, but only in the 65 and younger group of individuals (Levine *et al*., 2014). Because of the inefficient utilization of dietary protein associated with differences in protein quality (i.e., essential amino acid composition and digestibility), the minimum recommended intake, which has been set at 0.66 g/kg/day for men and women 18 years or older, is likely to be higher in the elderly compared to that in younger adults (Levine *et al*., 2014).

## Pharmacological interventions mimicking calorie restriction

### Inhibitors of the TOR pathway

The mTOR pathway has now been linked to lifespan and healthspan in several major model organisms and species. For instance, reduced mTOR signaling through genetic or pharmacological interventions leads to lifespan extension in yeast, worms, flies, and mice (Johnson *et al*., 2013), and studies are currently being conducted in primates and humans. Thus, mTOR signaling is a major candidate for targeted interventions. The mTOR kinase exists in two complexes: mTORC1 and mTORC2 (Laplante & Sabatini, 2012). Most studies indicate that reduced mTORC1 signaling confers longevity benefits (Johnson *et al*., 2013). However, individually reducing either mTORC1 or mTORC2 signaling extends worm lifespan (Vellai *et al*., 2003; Soukas *et al*., 2009). In addition to being responsive to insulin/IGF signaling, mTORC1 is activated by amino acids through the RAG GTPase complex and suppressed by stress signals or energy deficiency (Kim *et al*., 2013). In short, mTORC1 activation leads to protein translation and cell growth, whereas its inhibition blocks growth and induces stress response pathways such as autophagy (Laplante & Sabatini, 2012).

Many of the interventions that extend lifespan in model organisms have the effect of reducing mTORC1 signaling (Johnson *et al*., 2013; Kennedy & Pennypacker, 2014). These include protein and calorie/dietary restriction and reduced insulin/IGF signaling, as well as activation of AMP kinase and possibly of sirtuins, raising the question of whether the benefits of these interventions are, at least in part, dependent on their effects on mTORC1. Another fundamental question involves the downstream pathways of mTORC1 that mediate the longevity effects. Two major substrates of mTORC1, S6 kinase and 4E-BP1, are both linked to longevity. Loss of S6 kinase promotes longevity in yeast, flies, worms, and mice (Johnson *et al*., 2013), and increased 4E-BP1 activity promotes longevity at least in flies. In *C. elegans,* the DAF-16/FOXO transcription factor is required for lifespan extension in S6 kinase-defective mutants (Seo *et al*., 2013), as is autophagy (Hansen *et al*., 2008). Interestingly, DAF-16/FOXO and autophagy are both required for impaired insulin/IGF-1 signaling to extend lifespan in worms (Kenyon *et al*., 1993; Melendez *et al*., 2003), further linking these two perturbations.

An appealing aspect of considering mTOR as a target for anti-aging interventions is the availability of rapamycin, a pharmacological agent that is a specific inhibitor of mTOR, and that has been shown to extend lifespan in mice, up to 30% in females at high doses and to a lesser extent in males (Harrison *et al*., 2009; Miller *et al*., 2014). Rapamycin is not an active site inhibitor, but rather creates a trimolecular complex between the mTOR kinase and an FKBP protein, most notably FKBP12 (Brown *et al*., 1994; Sabatini *et al*., 1994; Marz *et al*., 2013). This action disrupts the mTORC1 complex leading to acute inhibition and prevents newly synthesized mTOR from entering either mTORC1 or mTORC2, leading to chronic inhibition of both complexes (Sarbassov *et al*., 2006).

Rapamycin has been studied for decades, tested in hundreds of clinical trials, and approved for use in several clinical conditions, including kidney cancers (Lamming *et al*., 2013; Kennedy & Pennypacker, 2014). However, rapamycin has important side effects, which limits its consideration as an anti-aging therapy. The adverse effects of rapamycin include metabolic dysregulation (e.g., hyperglycemia, hyperinsulinemia, and insulin resistance), proliferative defects in hematopoietic lineages, and others (Soefje *et al*., 2011). The metabolic side effects of rapamycin have been attributed to inhibition of mTORC2 (Lamming *et al*., 2012), suggesting that it may be possible to enhance efficacy-to-adverse effect ratios by specifically inhibiting mTORC1. Safety studies using rapamycin have been not been conducted in healthy people, so it is not clear whether rapamycin is harmful in healthy adults. Currently, several trials testing rapamycin are ongoing in healthy older populations, which will help evaluate the importance of mTOR in human aging and determine whether mTOR should be considered a target for interventions to extend healthspan.

In summary, ongoing studies to address the role of mTOR in aging and to determine whether it is a valuable target for interventions to extend healthspan are extensive and will likely shed light on these important questions in the near future.

### Inhibitors of glycolysis

Another area that is being actively explored as a strategy of mimicking DR is the inhibition of enzymes within the glycolytic pathway (Minor *et al*., 2009). It has been suggested that this strategy could provide a more effective upstream targeting to produce DR-like effects rather than targeting single downstream pathways. The rationale is that a greater cascade of responses could be activated, although this could also lead to a broader range of adverse effects (Minor *et al*., 2009). The first candidate drug in this category, 2-deoxy-D-glucose (2DG), which inhibits phosphoglucose isomerase, produced a remarkable phenotype similar to DR when fed to rats (Minor *et al*., 2009); however, other long-term studies reported that the concentrations necessary to elicit DR-like physiological responses also had cardiotoxic effects (Ganapathy-Kanniappan & Geschwind, 2013).

Recent preliminary studies have reported promising results with a nutraceutical product made from unripened avocados containing a seven-carbon sugar, mannoheptulose, which inhibits hexokinase (Minor *et al*., 2009). Studies in mice and dogs showed that the avocado extract improved insulin sensitivity as well as increased median lifespan in nematodes and mice. Additionally, there is currently great interest in applying glycolytic inhibitors in the treatment of cancer, as many tumor cells switch their metabolism to glucose as the major energy source (Ingram & Roth, 2010).

Mice with dysfunctional telomeres and senescent cells in culture display increases in energy demand and defects in mitochondrial biogenesis (Passos *et al*., 2010). In this context, supplementation with glucose improves tissue and body weight maintenance, increasing their lifespan (Missios *et al*., 2014). These results may appear counterintuitive as they suggest that an increase in dietary glucose can help maintain energy and tissue homeostasis at advanced age. Of note, however, malnutrition is present in more than 30% of geriatric patients. Furthermore, this requirement for additional glucose at older ages is analogous to that described above for protein, underscoring the need for a better understanding of optimal age-specific dietary composition and intake.

### Inhibitors of the GH/IGF-1 axis

Although the lack of global IGF-1 signaling is lethal, data from studies conducted in animal models have shown that a reduction in IGF-1 levels or IGF-1 action can extend lifespan. Additionally, human IGF-1 receptor gene polymorphisms are associated with exceptional longevity (Suh *et al*., 2008), and recently, Barzilai and colleagues have shown that low plasma IGF-1 concentrations predict survival in long-lived people (Milman *et al*., 2014), specifically in women with a history of cancer. In animals, dwarf, long-lived mice lacking the growth hormone receptor (GHR^-/-^) have reduced levels of IGF-1, are insulin sensitive despite obesity, and have decreased risk for cancer and diabetes (Zhou *et al*., 1997; Shevah & Laron, 2007; Ikeno *et al*., 2009). Importantly, similar results have been reported in growth hormone receptor-deficient Laron syndrome (LS) patients. In this regard, no formal aging studies have been performed on patients with LS; however, they are protected from diabetes and fatal neoplasms. (Guevara-Aguirre *et al*., 2011; Steuerman *et al*., 2011). Thus, pharmaceutical interventions that directly lower IGF-1 levels in adults could improve health and prolong lifespan.

Pharmacological targets for lowering IGF-1 action include those that act directly or indirectly on cells/tissues that produce or respond to GH and/or IGF-1. In this regard, human or humanized monoclonal antibodies and drugs directed against the IGF-1R have been used in clinical trials to treat several types of cancer (Warshamana-Greene *et al*., 2005; Carboni *et al*., 2009); however, none have been approved for clinical use. We are unaware of the development of any antibody against GH or IGF-1, but several classes of compounds that inhibit the GH/IGF-1 axis have been approved for use in patients with acromegaly. Recently, a consensus document has been developed for the use of this therapeutics (Giustina *et al*., 2014). One of these drug classes, somatostatin analogues, lower serum GH levels by suppressing GH secretion by pituitary somatotrophs, thereby ultimately decreasing serum IGF-1 levels. Unfortunately, these compounds also suppress secretion of other endocrine hormones, including insulin. Furthermore, only 20-50% of patients with acromegaly respond to these drugs, and significant adverse events have been documented including gallstones, diarrhea, and anorexia. Thus, the use of somatostatin analogues to increase longevity or healthspan appears to be unwarranted at this time.

The second approved drug for treating acromegaly is the GH receptor antagonist pegvisomant (Trainer *et al*., 2000; van der Lely *et al*., 2001; Kopchick *et al*., 2002; van der Lely & Kopchick, 2006). Pegvisomant is unique in that it does not inhibit GH secretion, but rather inhibits GH action by binding to and blocking the GHR (Kopchick *et al*., 2002). Notably, a dose-dependent decrease of IGF-1 levels is seen in up to 90% of pegvisomant-treated patients (Trainer *et al*., 2000; van der Lely *et al*., 2001; Kopchick *et al*., 2002; van der Lely & Kopchick, 2006). Additionally, pegvisomant is an insulin sensitizer that blocks the diabetogenic action of GH and thus produces beneficial effects on glucose metabolism. Pegvisomant, therefore, could have positive effects on both longevity and healthy aging by lowering serum IGF-1 and increasing insulin sensitivity. Regarding adverse effects, van der Lely *et al*. (2012)reported that *Long-term data on the efficacy and safety profile of pegvisomant are reassuring and few long-term serious adverse events have been reported but ongoing vigilance is required to monitor liver function and tumor size*. Thus, pegvisomant is an approved drug that should be tested for its effects on longevity and healthy aging. Future therapeutics targeted at inhibiting the GH/IGF-1 axis could include small inhibitory RNAs directed against the GHR or IGF-1 receptor mRNAs, monoclonal antibodies directed against GH or IGF-1, or novel GHR or IGF-1R tyrosine kinase inhibitors.

Another way to reduce global IGF-1 action may be to inhibit IGF-1 availability. For example, loss of PAPP-A, a protease that cleaves and inactivates the IGF-1 sequestering protein IGFBP-4, reduces IGF-1-induced signaling without affecting overall serum IGF-1 levels and not only extends mouse lifespan, but has many other beneficial effects on healthspan and age-related diseases (Conover, 2012).

In summary, reducing the activity of the GH/IGF-I somatotrophic axis is perhaps the most validated and consistent genetic intervention to extend mouse lifespan and healthspan. In addition GHR/IGF-I deficiency is also among the few phenotypes that is well characterized in humans (patients with Laron syndrome) with very few side effects in adults, even considering the extreme level of GH receptor deficiency and the resulting >80% reduction in circulating IGF-I. Notably, a pharmaceutical intervention targeting this pathway may or may not be designed to achieve such a low level of hepatic IGF-I secretion.

### Activators of the sirtuin pathways

The deacetylases known as sirtuins (SIRT1 to 7) promote longevity in diverse species and could mediate many of the beneficial effects of DR (Satoh *et al*., 2013). Given their apparent role in mediating health benefits, sirtuins have attracted considerable interest as a drug target. The first potent sirtuin-activating compounds (STACs) to be identified included several classes of plant-derived metabolites, such as flavones, stilbenes, chalcones, and anthocyanidins. These phytochemicals directly activate SIRT1 *in vitro* through an allosteric mechanism that lowers substrate *K*_m_ (Howitz *et al*., 2003). Resveratrol (3,5,4′-trihydroxystilbene) is still the most potent of these natural activators identified to date. The discovery of natural STACs prompted the production of synthetic SIRT1 activators that are considerably more potent, soluble, and bioavailable (Hubbard & Sinclair, 2014). In many studies, resveratrol and synthetic STACs have been shown to induce physiological and gene expression changes that are similar to DR, and improve function and extend the lifespan of numerous organisms including *S. cerevisiae*, *C. elegans*, *D. melanogaster*, *N. furzeri* (a short-lived fish), and *A. mellifera* (Hubbard & Sinclair, 2014).

An alternative approach to activating sirtuins, which raises the activity of the entire family of enzymes, is to exploit their common requirement for NAD^+^. NAD^+^ levels can be increased by providing NAD precursors (NMN or NR), by activating NAD biosynthetic enzymes (Wang *et al*., 2014a), or by inhibiting the NAD hydrolase CD38 (Yoshino *et al*., 2011; Canto *et al*., 2012; Escande *et al*., 2012; Gomes *et al*., 2014).

In mice, resveratrol extends lifespan when given to animals on a high-fat diet (Pearson *et al*., 2008) and in combination with standard chow when fed every other day (Baur *et al*., 2006; Pearson *et al*., 2008), but not when provided daily with standard chow (Pearson *et al*., 2008; Strong *et al*., 2013). Synthetic activators SRT1720 and SRT2104 also extend the lifespan of mice fed either a high-calorie or a low-calorie diet, and both protect mice against age-related changes in multiple tissues including muscle loss (Minor *et al*., 2011). In preclinical models, STACs have also shown considerable promise in treating diseases and complications associated with aging including cancer, type 2 diabetes, inflammation, cardiovascular disease, stroke, and hepatic steatosis (Hubbard & Sinclair, 2014).

Considerable progress has been made in the use of STACs to treat inflammatory and autoimmune disorders (Hubbard & Sinclair, 2014). For example, SRT1720 has beneficial effects in mouse models of chronic obstructive disease and asthma. In rhesus monkeys fed a high-fat, high-sugar diet, resveratrol exerts anti-inflammatory effects in visceral white adipose tissue (Jimenez-Gomez *et al*., 2013). STACs could also be beneficial in neurodegeneration (Zhao *et al*., 2013) based on mouse models of Alzheimer’s or Parkinson’s disease and multiple sclerosis (Graff *et al*., 2013; Hubbard & Sinclair, 2014). STACs prevent and reverse the effects of obesity and age-related metabolic decline. In mice fed a high-calorie diet, resveratrol and STACs protect against obesity, increase insulin sensitivity, increase mitochondrial function, and prevent liver steatosis (Baur & Sinclair, 2006; Baur *et al*., 2006). These effects are also seen in nonhuman primates fed a high-fat, high-sugar diet (Fiori *et al*., 2013; Jimenez-Gomez *et al*., 2013). Data from studies conducted in mouse models of obesity and aging have demonstrated that increasing NAD^+^ levels protects mice from metabolic decline and aging (Yoshino *et al*., 2011; Canto *et al*., 2012; Escande *et al*., 2012) and reverses mitochondrial decline, inflammation, and markers of muscle wasting (Gomes *et al*., 2014).

A considerable amount of data has accumulated on treating humans with STACs (Hubbard & Sinclair, 2014). Resveratrol has had mixed efficacy, in that its insulin-sensitizing effects (Ghanim *et al*., 2011; Smoliga *et al*., 2011) and DR-like phenotypes have been observed in elderly and obese humans (Timmers *et al*., 2011), but not in nonobese subjects with normal glucose tolerance (Yoshino *et al*., 2012). This suggests that STACs may restore homeostasis preferentially in metabolically compromised individuals. A meta-analysis of six unique datasets, including a total of 196 patients with type II diabetes (104 resveratrol, 92 control/placebo) showed statistically significant (*P* < 0.05) positive effects of resveratrol supplementation compared to placebo/controls for systolic blood pressure, hemoglobin A1c, and creatinine, but not for fasting glucose, homeostatic model assessment of insulin resistance, diastolic blood pressure, insulin, triglycerides, LDL, or HDL cholesterol (Hausenblas *et al*., 2014). Synthetic STACs have been tested in humans and are currently in clinical development (Venkatasubramanian *et al*., 2013; Hubbard & Sinclair, 2014). Human clinical studies with NAD^+^ precursors are expected in the next couple of years.

In summary, sirtuins and sirtuin activators have been implicated in longevity extension as well as in the prevention and treatment of a wide range of diseases in rodent models. However, there was not full consensus among meeting participants about the ability of sirtuin activators to slow aging, given long-running controversies in this area (Ledford, 2010; Couzin-Frankel, 2011).

## Activators of the AMPK pathway

AMP-activated protein kinase (AMPK) is a conserved, energy-sensing serine/threonine kinase that is activated when cellular energy levels are low, resulting in increasing levels of AMP (Ruderman & Prentki, 2004). AMPK activation generates insulin-sensitizing effects resulting in increased glucose uptake in skeletal muscles, and decreased hepatic glucose production and enhanced fatty acid oxidation in several tissues (Ruderman & Prentki, 2004; Ruderman *et al*., 2013). Activators have been developed, such as 5-aminoimidazole-4-carboxamide riboside (AICAR), and some FDA-approved drugs such as biguanides, thiazolidinediones, glucagon-like peptide-1 receptor agonists, salicylates, and resveratrol have AMPK-activating properties (Coughlan *et al*., 2014).

The biguanide, metformin, which is a first line therapy for type 2 diabetes mellitus (T2DM) activates AMPK in the liver (Rena *et al*., 2013). Evidence from animal models and *in vitro* studies suggests that metformin changes metabolic and cellular processes (Cabreiro *et al*., 2013) associated with the development of age-related conditions (Ruderman & Prentki, 2004). Notably, treatment of rats (Anisimov, 2010) mice (Martin-Montalvo *et al*., 2013) and nematodes (Cabreiro *et al*., 2013) with metformin extends lifespan. Exercise also stimulates AMPK and causes stimulation of glucose uptake and mitochondrial biogenesis during and after exercise. AICAR administration closely mimics these effects (Hayashi *et al*., 1998; Song *et al*., 2002).

Numerous studies are now focused on whether anti-aging effects of metformin can be demonstrated in patients with T2DM. Notably, in the United Kingdom Prospective Diabetes Study (UKPDS), metformin use, compared with other antidiabetic drugs, decreased the risk of cardiovascular disease (Group, 1998), cancer incidence and overall mortality (Wu *et al*., 2014), and possibly cognitive decline (Ng *et al*., 2014). Safety has been established and the pro-longevity effects noted would argue for testing the use of metformin in humans. However, to consider metformin for clinical trials testing its potential anti-aging effect, it will be important to closely examine the known effects of long-term use on a wide range of subjects and particularly those who are relatively healthy, but also those with specific conditions for whom chronic metformin use may have been shown to be detrimental. For example, recent evidence indicates that metformin also promotes inhibition of mitochondrial glycerophosphate dehydrogenase and gluconeogenesis, suggesting that only a partial understanding of the mechanisms of action of this powerful drug is known, and that further studies are necessary to determine whether it should be considered for treating generally healthy or relatively healthy populations (Madiraju *et al*., 2014). In fact, gluconeogenesis is required during fasting but also plays an important role in people consuming ketogenic diets, which may potentially make metformin dangerous for a subset of the population on diets that are relatively common and depend on gluconeogenesis.

## Inhibitors of inflammatory pathways

Chronic, low-grade inflammation is recognized as a major characteristic of aging. This phenomenon is so pervasive that the term *inflammaging* (Franceschi *et al*., 2000; Franceschi & Campisi, 2014) has been coined to emphasize that many major age-related disabilities, including cancers, susceptibility to infections, and dementia have immunopathogenic components (Franceschi & Campisi, 2014). Thus, as inflammation is associated with many age-related conditions, genes and pathways that regulate inflammation are candidate targets to combat them (Franceschi & Campisi, 2014). Inflammaging appears to be much more complex than we previously thought, and a variety of tissues and organs participate in producing inflammatory stimuli (Franceschi *et al*., 2007; Cevenini *et al*., 2012). The list is extensive and includes the immune system, but also adipose tissue, skeletal muscle, liver, and the gut. The gut is of unique importance, because it is the body’s largest immune organ and contains trillions of bacteria that can release inflammatory stimuli into the portal and systemic circulation (Biagi *et al*., 2010).

Despite its importance, mechanistic details for the most important stimuli that trigger inflammaging remain unknown, and much additional investigation is needed. Stimuli triggering inflammation can be exogenous (e.g., persistent cytomegalovirus infection) (Sansoni *et al*., 2014), but most are probably endogenously produced, possibly coming from the ‘self-debris’ resulting from the continuous turnover of cells and tissues (Franceschi & Campisi, 2014). For example, circulating mitochondrial DNA (mtDNA) is recognized by immune sensors as a foreign nucleic acid, is a powerful inflammatory stimulus, and increases with age (Pinti *et al*., 2014). Pro-inflammatory galactosylated N-glycans, which also represent one of the most powerful biomarkers of biological age in humans (Dall’Olio *et al*., 2012), and pro-inflammatory circulating microRNA (‘inflammaMIR’) (Olivieri *et al*., 2013) also increase in circulation with age and could contribute to inflammaging.

The most important drivers of age-dependent inflammation probably lie at the cellular and molecular levels. Cellular senescence is associated with a pro-inflammatory senescence-associated secretory phenotype (SASP), which is triggered by damaging agents (radiation, viruses) and possibly by continuous exposure to cellular debris (Coppe *et al*., 2010). Cellular senescence can also spread to neighboring cells (Jurk *et al*., 2014). Second, DNA and telomere damage caused by reactive oxygen species and other agents can trigger an inflammatory DNA damage response (Vitale *et al*., 2013). Third, activation of inflammasomes and the NF-kB pathway can be elicited by ROS and cellular debris (Youm *et al*., 2013). These mechanisms provide many targets for therapies to decrease inflammaging either locally or systemically. Possible strategies to target them include elimination of senescent cells (e.g., by NK cell activation)(Tchkonia *et al*., 2013), de-activation of inflammasomes (Youm *et al*., 2013), diets enriched with omega3 fatty acids (the ‘Mediterranean diet’)(Berendsen *et al*., 2013), and other nutritional strategies, such as DR discussed above.

In mouse studies, anti-inflammatory drugs showed a potential for extending lifespan, although their effects were relatively small (Strong *et al*., 2008); for example, nordihydroguaiaretic acid (NDGA) and aspirin increased survival but did not extend maximum lifespan. Other anti-inflammatory drugs were not effective in lifespan extension, underlining the need for additional and larger studies to determine the potential of NSAID and other anti-inflammatory drugs in extending human healthspan.

## Modulators of epigenetic pathways

The term epigenetics denotes heritable phenotypic alterations caused by postreplicative modifications of chromatin, rather than classical mutation-based genetic changes. Such covalent and noncovalent modifications of DNA and proteins (e.g., histones) alter the state of chromatin conformation and elicit corresponding changes in transcriptional activity (Jaenisch & Bird, 2003; Goldberg *et al*., 2007; Baker *et al*., 2008). Epigenetic effects can be elicited by three distinct principal means: (i) DNA methylation; (ii) post-translational histone modifications; and (iii) noncoding RNA interference (Goldberg *et al*., 2007; Baker *et al*., 2008). Twin studies suggest that genetics at birth determines only 25% of lifespan; therefore, it is proposed that epigenetic factors also contribute to aging. Such epigenetic factors are likely influenced by lifestyle, diet, and exogenous stress, raising the possibility that strategies can be developed to ameliorate age-associated cellular dysfunction (Imai *et al*., 2000; Longo, 2009).

Although manipulation of enzymes (sirtuins, histone acetyltransferases, histone deacetylases) that regulate the (de)acetylation status of chromatin (and other targets) can prolong lifespan in yeast, flies, and worms, the role of histone modifications in lifespan regulation is poorly understood. A fly model has recently been introduced in which the impact of such histone mutations on aging and lifespan can be evaluated (Pengelly *et al*., 2013).

A naturally occurring polyamine, spermidine, directly inhibits histone acetyltransferases (HATs), thereby maintaining histone H3 in a hypoacetylated state (Eisenberg *et al*., 2009). Functionally, this results in higher resistance to heat and oxidative stress as well as markedly reduced rates of cell necrosis during aging in human and yeast cells. Strikingly, this mechanism extends chronological lifespan across species, including flies, nematodes, and human cells. These data support the existing body of knowledge regarding histone acetylation in lifespan maintenance, including the finding that deletion of sas2, encoding a histone acetyltransferase, extends the replicative lifespan in yeast (Dang *et al*., 2009). Sas2 antagonizes Sir2, a prominent histone deacetylase involved in aging, and its deletion stabilizes Sir2 levels in aging cells, thereby allowing a low basal level of acetylation on specific histone residues associated with longevity regulation (Raisner & Madhani, 2008). Finally, a simple way to change age-related histone acetylation consists of dietary strategies that deplete cellular acetyl CoA, the sole donor for acetylation reactions. Indeed, depletion of acetyl CoA has been recently shown to be sufficient for autophagy induction and lifespan extension, although it is not known whether these effects are dependent on epigenetic changes (Eisenberg *et al*., 2014; Marino *et al*., 2014).

In humans, only nontoxic natural substances such as spermidine or resveratrol, which lead to deacetylation of chromatin, should be considered for clinical testing (Morselli *et al*., 2011). As a caveat, mechanistic understanding of this strategy is highly challenging as the drugs could have many off-target effects and even at the epigenetic level, the integrated response of multiple histone sites might be needed to mediate anti-aging effects. However, data from mice and humans indicate that spermidine has the potential to be safe for testing its epigenetic-dependent and independent effects on human healthspan. In one human study, a polyamine-rich traditional Japanese food (fermented soybeans) showed significant enhancement of polyamine concentration in the blood of the participants without obvious adverse effects (Soda *et al*., 2009).

## Other promising potential drugs and drug targets

### β2-adrenergic receptor (β2AR) signaling

Chronic administration of β2AR agonists increases mortality and morbidity (Ho *et al*., 2010). Conversely, β2AR antagonists (β-blockers) decrease mortality after myocardial infarction and improve the health of individuals with heart failure (Bristow, 2000; Ellison & Gandhi, 2005). Oral administration of the β-blockers, metoprolol and nebivolol, beginning at 12 months of age, increased the mean and median lifespan of isocalorically fed male C3B6F1 mice by 10 and 6.4%, respectively (Spindler *et al*., 2013). Neither drug affected body weight or food intake, eliminating DR or altered energy expenditure as explanations for these effects. The drugs also extended *Drosophila* lifespan without affecting food intake. The effects of long-term administration of β-blockers on human healthspan need to be investigated further in mice and humans before they can be considered for anti-aging interventions in healthy individuals.

### Meso-nordihydroguaiaretic acid (NDGA)

NDGA is a lignin present at high concentrations in creosote bushes (V.E.Tyler, 1994). Oral administration of NDGA extends *Drosophila* and mouse lifespan (Spindler *et al*., 2014). Studies *in vitro* show that NDGA inhibits intercellular inflammatory signaling, tumor cell proliferation, insulin-like growth factor-1 (IGFIR) and HER2 receptor activation, and oxidative phosphorylation (Pardini *et al*., 1970; Lu *et al*., 2010). NDGA reduces weight in a dose-dependent manner without change in food consumption, suggesting it either decreases absorption or increases caloric utilization (Spindler *et al*., 2014) NDGA was not overtly toxic in mice, but was associated with increased liver, lung, and thymus tumors, as well as peritoneal hemorrhage (Spindler *et al*., 2014). Less toxic derivatives of NDGA should be explored as anti-aging therapeutics in preclinical trials (Meyers *et al*., 2009; Castro-Gamero *et al*., 2013), although the associations with toxicities make it an unlikely candidate for human healthspan interventions.

### Statins and angiotensin-converting enzyme (ACE) inhibitors

Statins (3-hydroxy-3-methylglutaryl coenzyme A (HMG-CoA) reductase inhibitors) reduce age-related heart arrhythmias (Ludman *et al*., 2009; Spindler *et al*., 2012) and mortality from multiple types of cancer (Zeichner *et al*., 2012; Nielsen *et al*., 2013). The health benefits of statins stem from reduced protein isoprenylation (Spindler *et al*., 2012) and reduced cholesterol biosynthesis (Ludman *et al*., 2009). Statins increase the lifespan and healthspan of *Drosophila* by decreasing protein isoprenylation (Spindler *et al*., 2012). ACE inhibitors are antihypertensives (Crowley *et al*., 2012) that reduce AT_1_R activity, thereby reducing mortality and morbidity secondary to myocardial infarction (Corvol *et al*., 2004; Gradman, 2009; Hoogwerf, 2010; Crowley *et al*., 2012), and mitigating adverse cardiac events in patients with congestive heart failure (Corvol *et al*., 2004; Gradman, 2009; Hoogwerf, 2010; Crowley *et al*., 2012). Combined oral administration of statins and ACE inhibitors extends mouse lifespan by approximately 9% without affecting serum cholesterol or food intake. However, monotherapy with either drug is not effective. Statins and ACE inhibitors are generally well tolerated, and together, they could increase the lifespan of normotensive, normocholesterolemic individuals. Further studies and the understanding of their mechanisms of action and potential effects on aging are necessary before they can be considered anti-aging drugs. Considering the very wide use of statins in many countries, we should be able to begin to investigate its broader healthspan effects on the relatively healthy populations being treated for mildly elevated cholesterol.

### Hexosamine pathway and glycobiology

The hexosamine pathway produces the metabolite uridinediphosphate N-acetylglucosamine (UDP-GlcNAC), which is the precursor for N-linked glycosylation in the endoplasmic reticulum, and O-linked glycosylation in cytosol and other compartments. Activation of this pathway through gain-of-function mutations in the enzyme GFAT-1 (glutamine fructose 6-phosphate amino transferase) results in excess UDP-GlcNAC, and extension of *C. elegans* lifespan (Denzel *et al*., 2014). Activation of the hexosamine pathway or upregulation of GlcNAC enhances several aspects of protein quality control, including proteasome activity, autophagy, and ER-associated degradation pathways, which collectively lead to alleviation of phenotypes in models of proteotoxic disease. In mice, ischemia/reperfusion of the heart triggers the ER stress response, with upregulation of GFAT as one of the consequences. GFAT upregulation or GlcNAC treatment was reported to protect against ischemic challenge (Wang *et al*., 2014b). Additionally, the related metabolite glucosamine has been shown to extend murine lifespan (Weimer *et al*., 2014). These studies point to the potential significance of the hexosamine pathway in regulating protein quality control and longevity. Previous work in this area also suggested that overactivation of the hexosamine pathway could produce diabetic-like symptoms (Hawkins *et al*., 1996). Thus, the activity and tissue responses of this pathway will need to be optimized to achieve beneficial effects. Interestingly, the long-lived naked mole rat harbors extensive hyaluronan glycoconjugates (comprised of GlcNAC and glucuronic acid subunits), thought to be protective against cancer and perhaps other age-related diseases (Tian *et al*., 2013). Glycoconjugates are also suggested to serve as predictive biomarkers of human aging (Dall’Olio *et al*., 2013). Clearly, the hexosamine pathway could provide novel targets to treat age-related disease and further work in this area is merited.

### DNA damage signaling

Studies in mice provided the proof of principle that the deletion of upstream DNA damage responses (Exo1-dependent end resection) and downstream DNA damage checkpoints (p21-dependent cell cycle arrest, Puma-dependent apoptosis) can prolong tissue maintenance and increase the lifespan of aging telomerase-deficient mice (Choudhury *et al*., 2007; Schaetzlein *et al*., 2007; Sperka *et al*., 2011; Wang *et al*., 2012). This is a potential anti-aging target as telomere dysfunction and DNA damage accumulate in aging human stem cells and tissues (Jiang *et al*., 2008). Another connection between DNA damage signaling, telomeres and aging discussed at the meeting was the noncanonical activation of DNA damage pathways (e.g., ATM) by mitochondrial ROS (Schroeder *et al*., 2013). This extends yeast chronological lifespan by epigenetically silencing subtelomeric transcription, suggesting that mitochondrial adaptive ROS signaling pathways could potentially be targeted to extend healthspan.

### Stem cells

During aging, adult tissue stem cells exhibit impairments in functionality and exponential increases in premalignant mutations driven by cell-intrinsic defects and alterations in the stem cell niche and the blood circulatory environment (Ju *et al*., 2007; Behrens *et al*., 2014). Emerging data indicate that the reversal of age-associated defects in stem cell stability and function could help to improve tissue maintenance and to prevent stem cell-derived carcinogenesis during aging (Patel & Demontis, 2014). Both stem cell-based interventions and other dietary and pharmacological interventions which induce stem cell-based regeneration and rejuvenation are likely to be very important for healthspan in the future, but are currently only beginning to be tested as modulators of lifespan in model organisms.

### Retrotransposable elements

Evidence from multiple model systems, including yeast, *Drosophila*, and more recently, mouse and human cell culture demonstrate that retrotransposable elements become active during cellular senescence and aging (De Cecco *et al*., 2013a,b; Sedivy *et al*., 2013). Active retrotransposition is mutagenic and potentially highly destabilizing to genomes. Given the importance of genome integrity as it relates to cancer and aging, this raises the interesting and novel possibility that activation of retrotransposition could contribute to some age-associated pathologies. Some nucleoside reverse transcriptase inhibitors currently used clinically to treat HIV infection, such as lamivudine or adefovir, block retrotransposition of several endogenous elements, including LINE1, the only known active retrotransposition family in human genomes (Dai *et al*., 2011). While the current reverse transcriptase inhibitors have adverse effects arguing against their long-term use in humans as an anti-aging intervention, if studies in mice show beneficial effects, new drugs could be developed specifically to target LINE1 elements.

## Conclusions

Accumulating scientific evidence from studies conducted in various organisms and species suggests that targeting aging will not just postpone chronic diseases but also prevent multiple age-associated metabolic alterations while extending healthy lifespan. A number of pathways affecting metabolism, growth, inflammation, and epigenetic modifications that alter the rate of aging and incidence of age-related diseases have been identified. Interventions with the potential to target these pathways safely and to induce protective and rejuvenating responses that increase human healthspan are becoming available. These include intermittent or prolonged fasting, mild CR combined with a low glycemic index diet and protein restriction, inhibition of the GH/IGF-I axis, inhibition of TOR–S6K signaling, and activation of sirtuins or AMPK. Additional pharmacological interventions such as treatments with metformin, acarbose, spermidine, statins, and β-blockers should also be evaluated. While not yet ready for human trials, novel strategies including drugs that affect epigenetic modifications or inhibit retrotransposition deserve additional research and attention. Given the logistical issues of clinical studies aimed primarily at prolonging lifespan or healthspan, the participants of the workshop concluded that initial trials should be first designed to treat age-related diseases and conditions (not specifically aging), and should start with smaller cohorts, relatively short time periods, and a primary focus on safety and tolerability. This approach is likely to provide early clues for especially promising potential candidates that would then merit lengthier or more detailed studies potentially focused on aging.

In agreement with the title of this workshop *Interventions to Slow Aging in Humans: Are We Ready?,* we, the members of this workshop, believe that the time has come not only to consider several therapeutic options for the treatment of age-related comorbidities, but to initiate clinical trials with the ultimate goal of increasing the healthspan (and perhaps longevity) of human populations, while respecting the guiding principle of physicians *primum non nocere*.
